# Genome-wide analysis of MADS-box gene family in kiwifruit (*Actinidia chinensis* var. *chinensis*) and their potential role in floral sex differentiation

**DOI:** 10.3389/fgene.2022.1043178

**Published:** 2022-11-17

**Authors:** Li-Xia Ye, Min-Min Luo, Zhi Wang, Fu-Xi Bai, Xuan Luo, Lei Gao, Jue Peng, Qing-Hong Chen, Lei Zhang

**Affiliations:** ^1^ Institute of Fruit and Tea, Hubei Academy of Agricultural Sciences, Wuhan, China; ^2^ College of Horticulture and Gardening, Yangtze University, Jingzhou, China

**Keywords:** genome-wide, MADS-box gene family, kiwifruit, flower, sex differentiation

## Abstract

Kiwifruit (*Actinidia chinensis* Planch.) is a functionally dioecious plant, which displays diverse morphology in male and female flowers. MADS-box is an ancient and huge gene family that plays a key role in plant floral organ differentiation. In this study, we have identified 89 MADS-box genes from *A. chinensis* Red 5 genome. These genes are distributed on 26 chromosomes and are classified into type I (21 genes) and type II (68 genes). Overall, type II AcMADS-box genes have more complex structures than type I with more exons, protein domains, and motifs, indicating that type II genes may have more diverse functions. Gene duplication analysis showed that most collinearity occurred in type II AcMADS-box genes, which was consistent with a large number of type II genes. Analysis of *cis*-acting elements in promoters showed that AcMADS-box genes are mainly associated with light and phytohormone responsiveness. The expression profile of AcMADS-box genes in different tissues showed that most genes were highly expressed in flowers. Further, the qRT-PCR analysis of the floral organ ABCDE model-related genes in male and female flowers revealed that *AcMADS4*, *AcMADS56*, and *AcMADS70* were significantly expressed in female flowers. It indicated that those genes may play an important role in the sex differentiation of kiwifruit. This work provided a comprehensive analysis of the AcMADS-box genes and may help facilitate our understanding of the sex differentiation regulatory mechanism in kiwifruit.

## 1 Introduction

The MADS-box is an ancient and very large gene family that widely exists in eukaryotes, such as yeast, plants, insects, and mammals, regulating growth and signal transduction (Ng and Yanofsky, 2001; Messenguy and Dubois, 2003). The MADS-box protein contains four conserved domains: MADS-box (M), Intervening (I), Kertain-like (K), and C-terminal (C) from N-terminal to C-terminal (Parenicova et al., 2003). The M domain is a highly conserved domain composed of 56–60 amino acids (aa) and located at the N-terminal (Theien and Gramzow, 2016). It can specifically recognize and bind to the CArG-box [CC (A/T)_6_ GGG] to participate in protein dimerization (Niels et al., 2018). The K domain has about 70 aa, which is less conservative than the M domain. Three α-helices (K1, K2, and K3) in the K domain form a coiled helix structure and participate in protein-protein interactions (Yang et al., 2003). The low-conservative I domain is composed of 30 aa and promotes protein dimers binding to DNA. The C domain is rich in hydrophobic amino acids with variable sequences and lengths and is primarily responsible for the formation of protein complexes and transcriptional activity (Theien and Gramzow, 2016). Based on the evolutionary relationship, the MADS-box gene family members can be divided into two categories: type I (M type) and type II (MIKC type) (Becker and Günter, 2003). Type II MADS-box gene has complete domains, while type I MADS-box gene lacks K domain (Parenicova et al., 2003). MIKC type can be further divided into MIKC* and MIKCC types according to the sequence characteristics of the I region. MIKCC type is further divided into 12 subgroups (*SEP*, *AGL6-like*, *AP1*, *FLC*, *SOC1*, *AGL15-like*, *AGL12-like*, *ANR1*, *SVP*, *PI*, *TT16*, and *AG*) based on sequence and function, and M type is further divided into Mα, Mβ, and Mγ subgroups. Type I and type II MADS-box genes have different evolutionary histories in various species (Kofuji et al., 2003). Type II MADS-box genes are mainly derived from whole genome duplication (Airoldi and Davies, 2012). In most species, the gene structure of type II MADS-box genes are more complex than type I, such as Arabidopsis (Kofuji et al., 2003), rice (Arora et al., 2007), maize (Zhao D. et al., 2021).

The MADS-box genes are involved in regulating many biological processes, such as seed germination (He et al., 2019), vegetative growth (Cohen et al., 2012), floral organ specification (Kong et al., 2022), flowering (Liu et al., 2022), embryo and fruit development (Colombo et al., 2008; Pi et al., 2021). At present, most of the floral organ development-related genes belong to the MADS-box family. In the ABCDE model that regulates the development of floral organs, except for *APETALA2* (*AP2*) in A-type, other A-type genes *AP1*, B-type genes *PISTILATA* (*PI*) and *AP3*, C-type gene *AGAMOUS* (*AG*), D-type genes *SEEDSTICK* (*STK*) and E-type genes *SEPALLATA1* (*SEP1*), *SEP2*, *SEP3*, and *SEP4* all belong to the MADS-box gene family (Liu et al., 2018). The ABCDE floral organ development model showed that A-type genes regulate sepal development, B-type genes regulate petal and stamen development, C-type genes regulate stamen and carpel development, D-type genes regulate ovule development, and E-type genes are expressed in petal, stamen, pistil and sepal primordium, affecting the development of the whole floral organ (Shen et al., 2019; Shen and Wang, 2022).

Kiwifruit is a functionally dioecious plant, which show distinct morphology between the flowers of female and male plants (Coimbra et al., 2004; Caporali et al., 2019). The male and female flowers are similar in the early stage of development. However, the pistil in male flowers stop developing shortly after the initiation of stigma development, while stamen development in female flowers continue to the later stage and produce nonviable pollen (Caporali et al., 2019; Varkonyi-Gasic et al., 2021). Two key sex determining genes, *Shy Girl* (*SyGl*) and *Friendly Boy* (*FrBy*), have been isolated and identified in kiwifruit (Akagi et al., 2019). *SyGl* is an *ARR22*/*24-like* C-type cytokinin response inhibitor, which is specifically expressed in male flower carpels and inhibits carpel development by regulating the cytokinin response pathway (Akagi et al., 2018; Caseys, 2018). The application of cytokinin can partially restore male carpel development (Varkonyi-Gasic et al., 2021). The male-promoting factor *FrBy* encodes a FAS1 structural protein and regulates the pollen fertility of female flowers by delaying apoptosis of anther tapetum cells (Akagi et al., 2019). However, the regulatory networks of these genes are still unknown. The ABCDE model-related genes have an important role in floral sex differentiation (Hui et al., 2017; Coito et al., 2018), and it may also be involved in regulating the differentiation of male and female flowers in kiwifruit.

Here, the AcMADS-box genes of kiwifruit were isolated, and the chromosome location, gene structures, *cis*-acting elements of the promoter, protein conserved domains, motifs, and different tissue expression profiles were analyzed. In addition, qRT-PCR analysis was performed on the floral organ ABCDE model-related genes in the different organs of kiwifruit male and female flowers. The results showed that *AcMADS4*, *AcMADS56,* and *AcMADS70* might be involved in the differentiation of male and female flowers in kiwifruit.

## 2 Materials and methods

### 2.1 Identification of AcMADS-box genes

The genomes of *A. chinensis* Red 5 and *Arabidopsis thaliana* were downloaded from the Kiwifruit Genome Database (KGD) (http://kiwifruitgenome.org) (Pilkington et al., 2018; Yue et al., 2020) and The Arabidopsis Information Resource (TAIR) (http://www.arabidopsis.org) (Garcia-Hernandez et al., 2002), respectively. The MADS-box protein sequences of *A. thaliana* (AtMADS-box) were isolated by the gene ID (Supplementary Table S2) reported by Zhao W. et al. (2021). First, local BLASTp of AtMADS-box protein sequences with the whole genome of *A. chinensis* Red 5 (Red5_pep_v1.0.fa.gz) were performed in TBtools to screen for candidate MADS-box proteins of *A. chinensis* (AcMADS-box). Subsequently, all candidate proteins were submitted to the NCBI Conserved Domain Database (CDD) (https://www.ncbi.nlm.nih.gov/cdd/) (Marchler-Bauer et al., 2015) to check the MADS and K-box domains, and the candidate proteins without MADS or K-box domains were removed. And then, to remove incomplete sequences, each candidate AcMADS-box gene was manually checked. Finally, a total of 89 AcMADS-box genes were identified.

The chromosome location informations of AcMADS-box genes were analyzed and visualized from the *A. chinensis* Red 5 genome annotation file (Red5_v1.0.gff3.gz) by Gene Location Visualize from GTF/GFF tool in TBtools (Chen et al., 2020). The 89 AcMADS-box genes were named from *AcMADS1* to *AcMADS89* according to the order of appearance from chromosome 1 to chromosome 29. The genomic DNA (gDNA) sequences and the coding sequence (CDS) were isolated from the *A. chinensis* Red 5 genome files (Red5_genome_v1.0.fa.gz, Red5_cds_v1.0.fa.gz) by Sequence Toolkit in TBtools (Chen et al., 2020). The physicochemical properties (isoelectric point, molecular weight, and instability index) of AcMADS-box genes were predicted on the ExPasy website (https://web.expasy.org/protparam/) (Wilkins et al., 1999), and their subcellular localization was predicted by BUSCA website (http://busca.biocomp.unibo.it/) (Savojardo et al., 2018).

### 2.2 Phylogenetic analysis and classification of AcMADS-box genes

To analyze the phylogenetic relationship and classification of AcMADS-box genes, the protein sequences of 89 AcMADS-box and 109 AtMADS-box were aligned using the ClustalW multiple method in BioEdit software, and then an unrooted phylogenetic tree for AcMADS-box and AtMADS-box proteins was constructed using the Maximum-Likelihood (ML) method with 1,000 bootstrap replicates in MEGA 11 (Tamura et al., 2021). The other parameters were default and the bootstrap values less than 50% were hidden. The AcMADS-box proteins were classified into subgroups according to their phylogenetic relationships with the corresponding AtMADS-box subgroup genes. The phylogenetic tree was beautified by the Adobe Illustrator tool (McLean, 2002).

### 2.3 Gene structure, conserved domain, motif, and *cis*-acting elements analysis

The gDNA, CDS, and protein sequence of AcMADS-box genes were isolated from the *A. chinensis* Red 5 genome. The exon-intron structural map of AcMADS-box genes was constructed based on gDNA and CDS sequence alignment by Gene Structure View Toolkit in TBtools (Chen et al., 2020). The number of exons were counted in the exon-intron structural map and listed in Supplementary Table S1. The conserved domains were blasted in the NCBI-CDD website. The motifs of AcMADS-box proteins were identified in the online software MEME (https://meme-suite.org.) (Bailey et al., 2015) with the following parameters: motif count is 20; motif width ranges from 5 to 50 amino acids. To analyze the *cis*-acting elements of promoter, 2 kb promoter sequences of AcMADS-box genes were isolated from the *A. chinensis* Red 5 genome files (Red5_genome_v1.0.fa.gz) by Sequence Toolkit in TBtools (Chen et al., 2020). And then the promoter sequences were submitted to the PlantCARE website (http://bioinformatics.psb.ugent.be/webtools/plantcare/html) (Lescot et al., 2002) for *cis*-acting element identification. Finally, the gene structure, conserved domains, motifs, and *cis*-acting element of AcMADS-box genes were both visualized by BioSequence Structure lllustrator toolkit in TBtools (Chen et al., 2020) and beautified by the Adobe Illustrator tool (McLean, 2002).

### 2.4 Gene duplication and synteny analysis

Firstly, the length and gene density informations on 29 chromosomes of kiwifruit were obtained from the *A. chinensis* Red 5 genome annotation file (Red5_v1.0.gff3.gz) by Fasta Stats and Table Row Manipulate tools in TBtools (Chen et al., 2020). Then, the chromosome locations and relative distances of 89 AcMADS-box genes were isolated by GXF Gene Position and Info. Extract tool in TBtools (Chen et al., 2020). Then, the multiple collinearity scan toolkit X (MCScanX) in TBtools was used to perform the collinearity scanning of AcMADS-box genes under the condition of default parameters. Finally, the gene duplication events were analyzed according to the collinearity data. The detailed information on all the collinearity of the AcMADS-box genes are shown in Supplementary Table S6. The circos map was drawn by Advanced Circos in TBtools (Chen et al., 2020) and beautified by the Adobe Illustrator tool (McLean, 2002).

### 2.5 Expression profiles analysis

Gene expression profiles of AcMADS-box genes was generated with NCBI-SRA RNA-seq libraries of root (SRR13413574), shoot (SRR13413573), cane (SRR13413553), source leaf (SRR13413575), sink leaf (SRR13413576), flower bud (SRR13413579), opening flower (SRR13413552), the fruit of 20 days after anthesis (DAA) (SRR13413578), 147 DAA (SRR13413556) and 224 DAA (SRR13413557). The data were aligned to the *A. chinensis* Red5 Genome by HISAT2 with the main parameter: —dta -p 6—max-intronlen 5000000 (Kim et al., 2015). After the alignment analysis was completed, the reads on the alignment were assembled and quantified using StringTie with the main parameter: —merge -F 0.1 -T 0.1 (Pertea et al., 2015). Integrated Genomics Viewer (IGV) (Thorvaldsdottir et al., 2013) was used to visualize the mapping output (BAM format) and annotation file of the reference genome. The number of mapped reads and the length of transcripts in the samples were normalized by FPKM to measure the gene expression level (Shahriyari, 2019). Finally, visualization of AcMADS-box gene expression level by HeatMap Illustrator tool inTBtools (Chen et al., 2020).

### 2.6 Expression analysis of ABCDE model-related AcMADS-box genes by quantitative Real-Time PCR

The flowers samples were collected from the male and female plants of the same hybrid generation which were 6 years old and grow very well in the National Kiwifruit Germplasm Resource Garden of China, located in the Research Institute of Fruit and Tea, Hubei Academy of Agricultural Sciences (30°29’ N, 114°16’ E), Wuhan, China. The male and female flowers were sampled at stage 4 when flowers begin to differentiate (Caporali et al., 2019), each sample containing three biological replicates. The sepals, petals, pistils, stamens, and ovaries of male and female flowers were separated and frozen in liquid nitrogen immediately, and then stored at −80°C. According to the manufacturer’s instructions, the total RNA was extracted by Total RNA kit (Aidlab Biotechnology, China) and the first strand cDNA was synthesized by SuperScript™ IV VILO™ Master Mix (Thermo Fisher, China). The qRT-PCR was performed in Applied Biosystems 7500 Real-Time PCR System with the reaction system as follows: 5 μL of Hieff qPCR SYBR Green Master Mix (YEASEN, Shanghai, China), 0.2 μL each for forward and reverse primers, 4.1 μL of ddH_2_O, and 0.5 μL cDNA. The 2^−ΔΔCT^ method was used to calculate the relative gene expression of AcMADS-box genes (Arocho et al., 2006). The *AcActin* gene (Acc05529.1) was used for normalization of qRT-PCR data. The primers are listed in Supplementary Table S9.

## 3 Results

### 3.1 Identification of kiwifruit MADS-box genes

**FIGURE 1:**
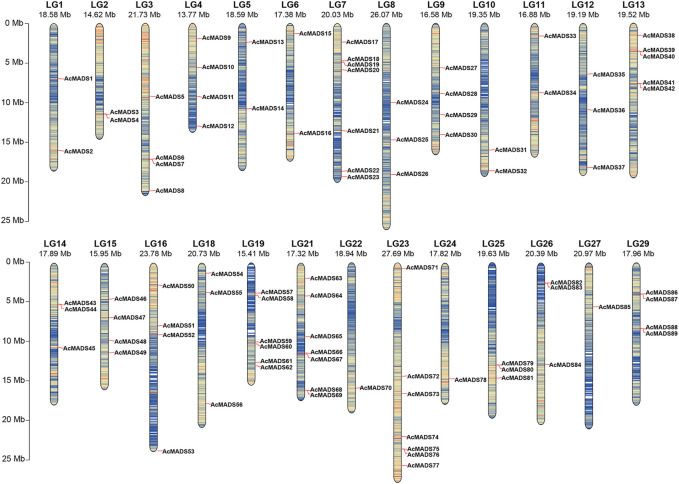
Chromosomal distribution of the *A. chinensis* MADS-box genes. LG17, LG20, and LG28 have no AcMADS-box gene distribution. The chromosome number is markered at the top, and the colors from blue to red on every chromosome indicate gene density. The chromosome length scale is located on the left.

Local BLASTp and HMM analyses were used to identify the kiwifruit MADS-box genes. After removing three candidate genes (Acc01231, Acc29205, and Acc30998) which without MADS or K-box domains, a total of 89 AcMADS-box genes were identified from the whole genome of *A. chinensis* Red 5. These AcMADS-box genes were renamed from AcMADS1 to AcMADS89 in the order of appearance from chromosomes 1 to 29 (Figure 1). The 89 AcMADS-box genes were distributed on 26 chromosomes of kiwifruit, with linkage group 7 (LG7), LG21, and LG23 having the largest number of genes, each of 7 AcMADS-box genes. However, no AcMADS-box gene was found on LG17, LG20, and LG28 (Supplementary Figure S1). Subsequently, the physicochemical properties of AcMADS-box proteins were analyzed, including the number of amino acids (aa), molecular weight (MW), theoretical isoelectric point (pI), instability index (II), and predicted subcellular localization (SL) (Supplementary Table S1). The length of AcMADS-box protein ranges from 62 (AcMADS88) to 499 aa (AcMADS24), and the MW varied from 7.17 (AcMADS88) to 56.40 kDa (AcMADS24). The pI analysis indicated that most AcMADS-box proteins are alkaline with pI > 7.5, 24 AcMADS-box proteins are acidic with pI < 6.5 and 9 AcMADS-box proteins are neutral with pI between 6.5 and 7.5. Additionally, 72 AcMADS-box proteins were unstable with II > 40, and only 17 AcMADS-box proteins were stable, such as AcMADS71 and AcMADS79. These results indicated that the length and molecular weight of AcMADS-box proteins are varied, and most AcMADS-box proteins are alkaline unstable proteins. The subcellular localization prediction of AcMADS-box genes showed that most AcMADS-box proteins may locate in the nucleus (75.28%), and the others located in the chloroplast (24.72%) (Supplementary Table S1). This is consistent with the regulatory role of transcription factors in the nucleus.

### 3.2 Phylogenetic analysis and classification of kiwifruit MADS-box genes

**FIGURE 2:**
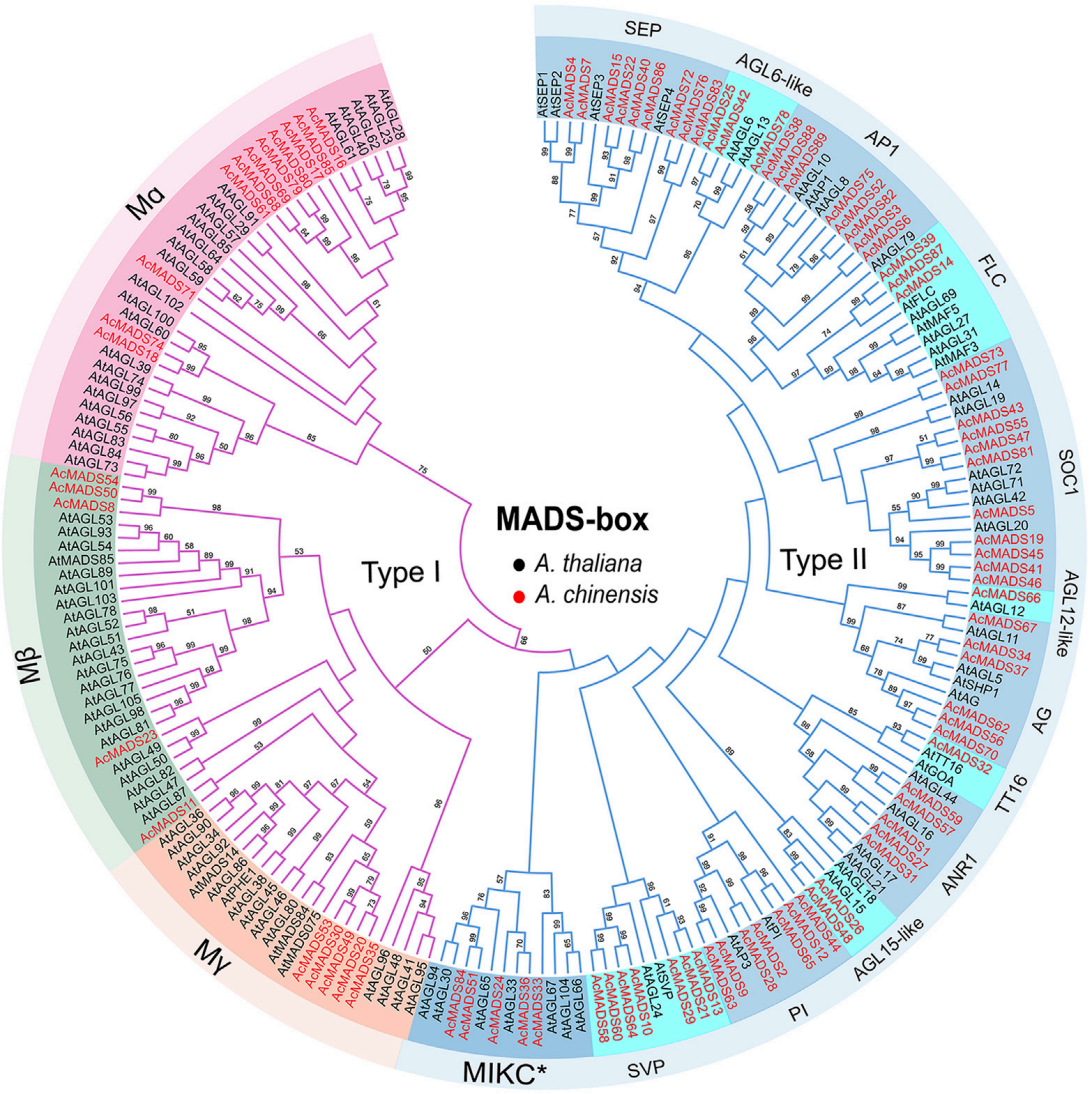
Unrooted Phylogenetic tree of *A. chinensis* and *A. thaliana* MADS-box genes. The MADS-box gene’s names are indicated with dark and red for *A. thaliana* (109) and *A. chinensis* (89), respectively. The purple and blue lines represent type I and type II MADS-box proteins. The names in the outside circle indicate different subgroups. The numbers above the line are bootstrap values, and the bootstrap values less than 50 are not listed.

To elucidate the evolutionary relationship of AcMADS-box genes, we aligned 89 AcMADS-box proteins from kiwifruit and 109 AtMADS-box proteins from Arabidopsis, and then constructed a phylogenetic tree (Supplementary Table S2, Figure 2). According to the evolutionary relationship, AcMADS-box genes were divided into two types: type I (21 genes) and type II (68 genes) (Figure 2). The 21 type I AcMADS-box genes were further subgrouped into Mα (11 genes), Mβ (5 genes), and Mγ (5 genes). The Mα group has the maximum AcMADS-box gene number of 11 in kiwifruit, which was consistent with the maximum members of 24 in Arabidopsis (Supplementary Table S2). The remaining 68 type II AcMADS-box genes were grouped into 13 subgroups (MIKC*, *SEP, AGL6-like, AP1, FLC, SOC1, AGL15-like, AGL12-like, ANR1, SVP, PI, TT16*, and *AG*) based on the classification of MIKCC type AtMADS-box genes (Figure 2). The statistical results showed that AcMADS-box were distributed in each subgroup (Supplementary Figure S2). The subgroups *AGL12-like* and *TT16* only have one AcMADS-box proteins. The subgroup *SOC1* have the maximum number of AcMADS-box proteins with 11, while the subgroup MIKC* has the maximum number of type II MADS-box proteins in Arabidopsis, which indicates that gene gain or loss events in the subgroup of AcMADS-box genes were occurred during kiwifruit evolution, and the *SOC1* subgroup may have an important role in kiwifruit.

### 3.3 Gene structure and protein domain analysis of MADS-box genes in kiwifruit

**FIGURE 3:**
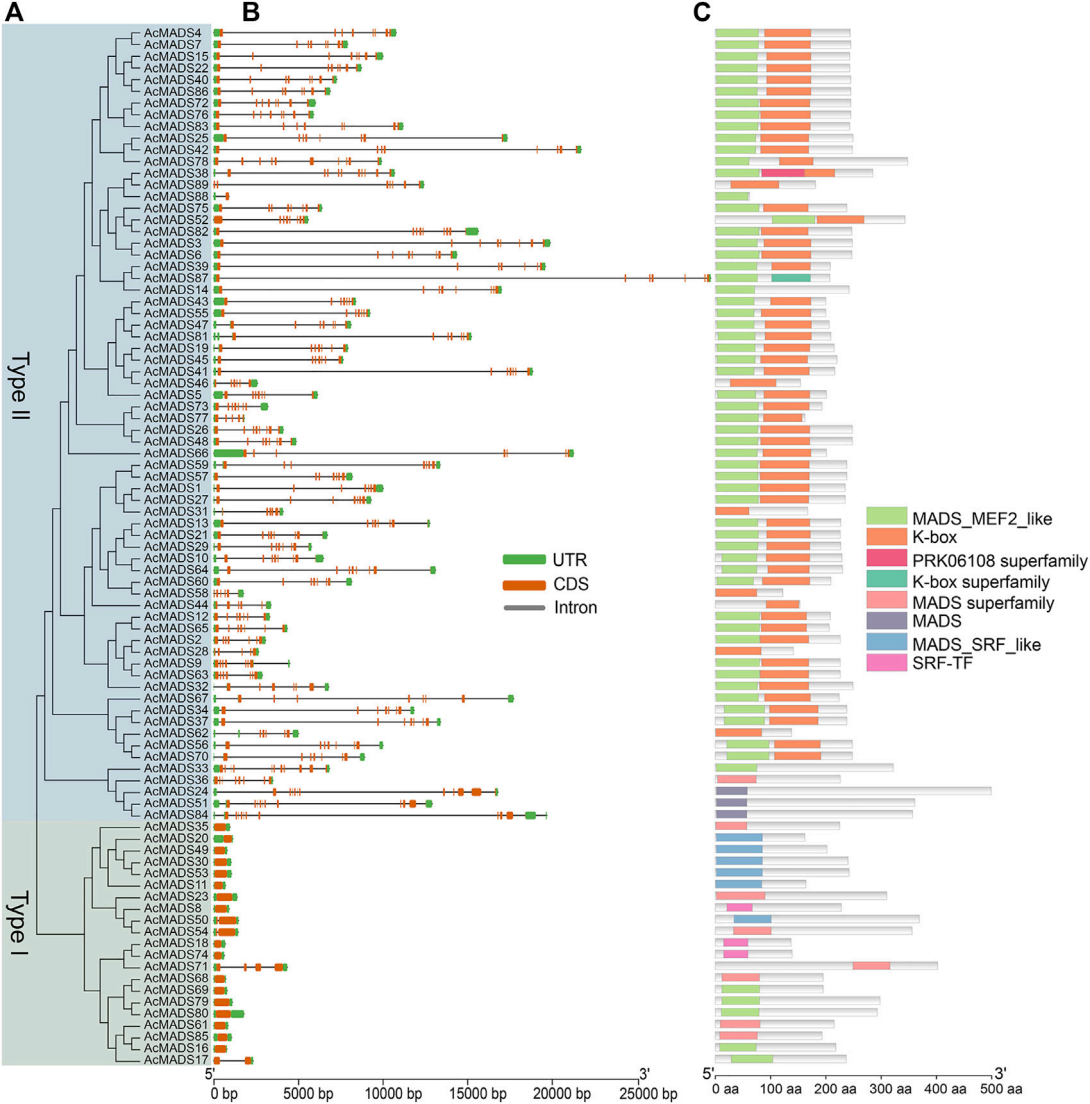
Phylogenetic relationships, gene structures, and conserved domains of MADS-box genes in *A. chinensis*. **(A)** The phylogenetic tree of 89 AcMADS-box proteins. **(B)** Gene structure of AcMADS-box genes. Green boxes represent UTR, orange boxes represent CDS, and gray lines represent introns. **(C)** The conserved domains of AcMADS-box proteins. The details of each domain are presented in Supplementary Table S3.

To obtain more information about the gene structures of 89 AcMADS-box genes, the gDNA and CDS sequences were analyzed by TBtools. In general, the gene structures of type II AcMADS-box are more complex (Figures 3A,B). Among the 21 type I AcMADS-box genes, 17 genes have only one exon, while only one type II gene has one exon, and the remaining 67 types II genes have over 5 exons. The type II AcMADS-box genes have a mean of 7.5 exons, and that in the type I was only about 1.3. The 4 MIKC* subgroup AcMADS-box genes (*AcMADS24, AcMADS33, AcMADS51*, and *AcMADS84*) have the largest number of exons (11) (Supplementary Table S1). In addition, the average length of gDNA, CDS and amino acid of type II AcMADS-box genes were about 9.59 kb (from 899 to 29385 bp), 0.70 kb (from 189 to 1500 bp) and 231 aa (from 62 to 499 aa), respectively, while that of type I AcMADS-box genes were about 1.23 kb (from 619 to 4347 bp), 0.72 kb (from 414 to 1209 bp) and 239 aa (from 137 to 402 aa) (Supplementary Table S1). The most obvious differences between two types AcMADS-box genes are DNA length and exon/intron structure, suggesting that type II AcMADS-box genes may form more types of proteins through alternative splicing.

To identify the conserved domains, the protein sequences of 89 AcMADS-box genes were submitted and predicted in CDD tool of NCBI. AcMADS-box proteins contained 8 types of conserved domains in three categories: MADS (MADS, MADS superfamily, MADS-MEF2-like, MADS-SRF-like, and SRF-TF), K-box (K-box, K-box superfamily), and PRKO6108 superfamily (Figure 3C). Usually, type I MADS-box proteins only contain the MADS domain, while type II proteins have both MADS and K-box domains (Parenicova et al., 2003). In kiwifruit, all type I AcMADS-box proteins contained MADS domain, and 3 of them (AcMADS8, AcMADS18, and AcMADS74) contained the SRF-TF type MADS domain (Supplementary Table S3). For 68 type II AcMADS-box proteins, 55 proteins contained both MADS and K-box domain, while 6 proteins only contained the MADS domain and 7 proteins only contained the K-box domain (Supplementary Table S3). These results suggested that some conserved domains of AcMADS-box proteins were lost during evolution. Noteworthy, only the AcMADS38 protein contained three domains (MADS, K-box, and PRKO6108 superfamily domain) (Figure 3C), which indicated it may gain some special function in kiwifruit.

### 3.4 Protein motif analysis of kiwifruit MADS-box genes

**FIGURE 4:**
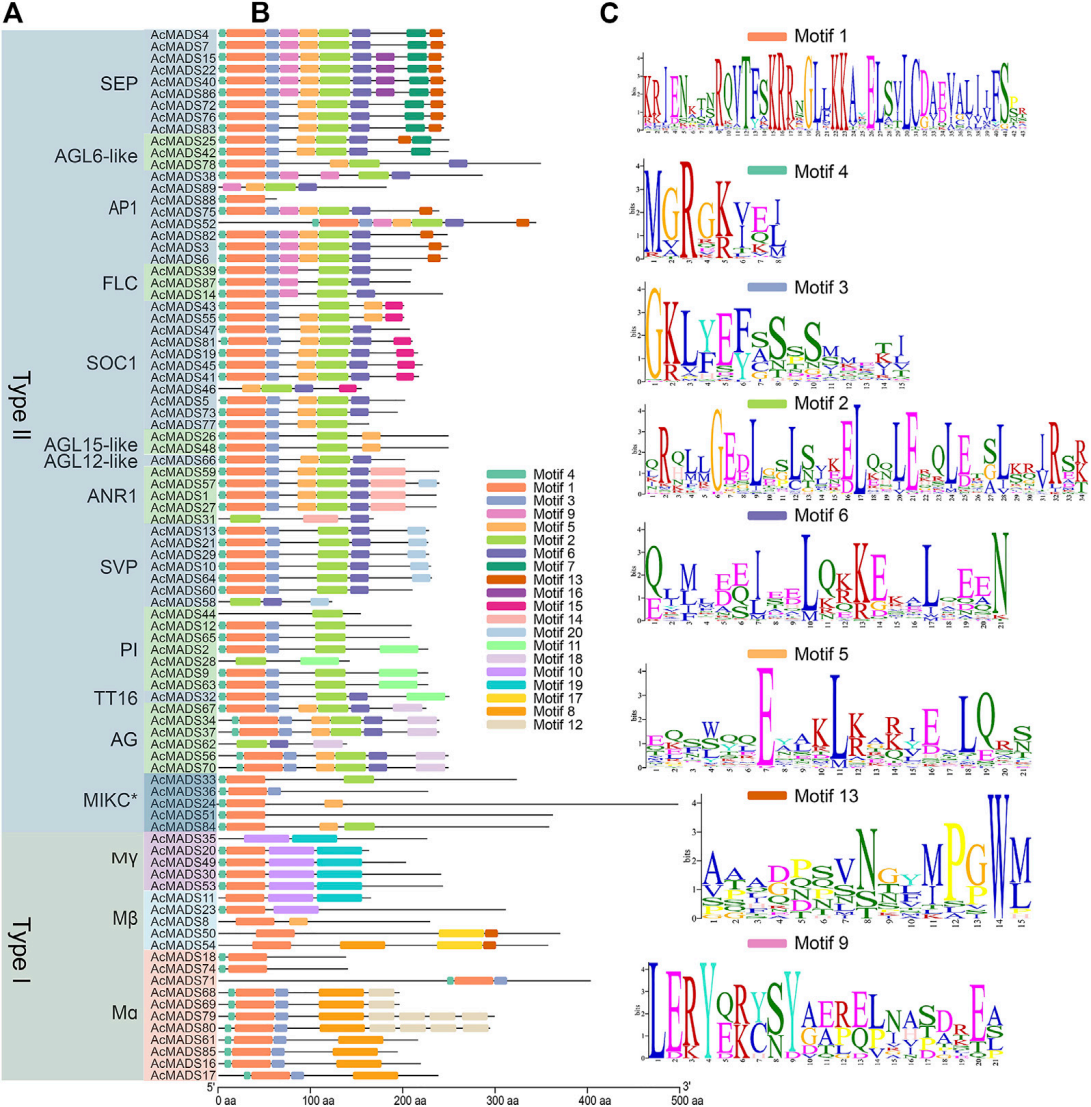
Protein motifs of MADS-box genes from *A. chinensis*. **(A)** The phylogenetic tree of 89 AcMADS-box proteins. **(B)** Motif locations of the 89 AcMADS-box proteins. The 20 types of motifs are shown with different colors boxes. **(C)** Sequence logos of the top 8 motifs with the largest number of genes. The details of each motif are listed in Supplementary Table S4.

To further explore the functional differentiation of the 89 AcMADS-box genes, 20 conserved motifs from motifs 1 to 20 of the AcMADS-box genes were identified and analyzed by the MEME website and TBtools (Figure 4). The lengths of the motifs ranged from 8 to 50 aa (Figure 4C and Supplementary Table S4). Motif 1 and 4 are highly conserved and distributed in almost all members of the AcMADS-box family. Motif one was found in 80 AcMADS-box proteins, and Motif 4 was found in 77 AcMADS-box proteins (Supplementary Table S4). However, some motifs are only found in specific subgroups, such as motif 2, 6, 7, 9, 11, 14, 15, 16, 18, and 20 specifically appear in type II AcMADS-box proteins, and motif 8, 10, 12, 17, and 19 are only present in type I AcMADS-box proteins (Figures 4A,B). The motif 12, 14, 15, 16, 17, and 18 are unique to Mα, *ANR1*, *SOC1*, *SEP*, Mβ, and *AG* subgroups, respectively (Figures 4A,B), which may cause gene function differentiation. Overall, most proteins in the same subgroup contain similar motifs, such as *FLC*, *AGL15-like*, and *AG* subgroups (Figures 4A,B), which indicated that AcMADS-box proteins of the same subgroup have the identical motif pattern and may have similar functions. However, some motifs of the AcMADS-box proteins, such as AcMADS28, AcMADS31, AcMADS35, AcMADS58, and AcMADS62 are different from other members of the same subgroup (Figures 4A,B), which may enrich the gene function of the same subgroup.

### 3.5 Promoter cis-acting elements analysis of kiwifruit MADS-box genes

**FIGURE 5:**
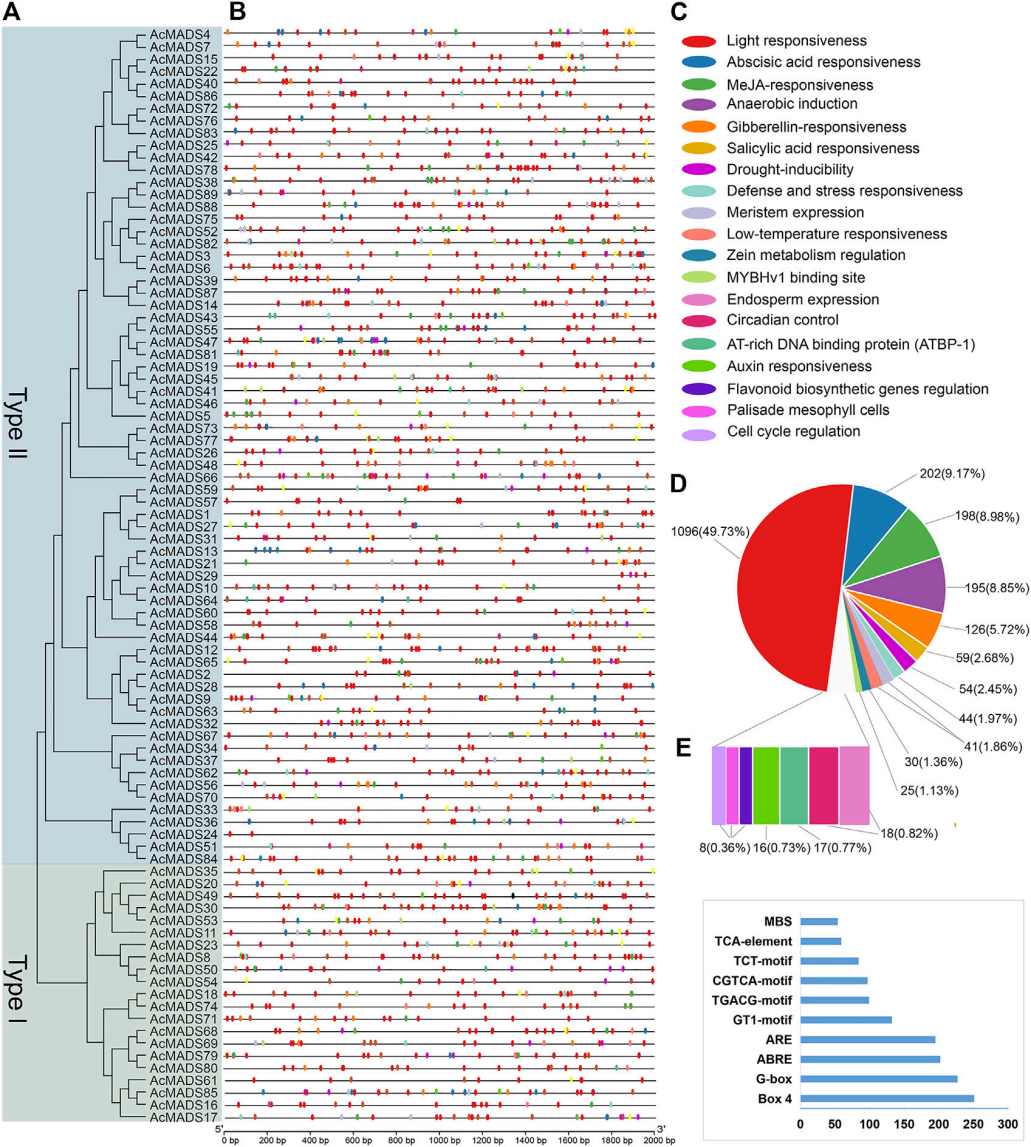
*Cis*-acting elements of AcMADS-box gene promoter from *A. chinensis*. **(A)** The phylogenetic tree of 89 AcMADS-box proteins. **(B)** The locations of *cis*-acting elements in the promoter of 89 AcMADS-box genes. **(C)** The 19 types of functional modules are shown as flat round boxes of different colors. **(D)** Statistical analysis of the 19 types of functional modules in *cis*-acting elements. **(E)** Statistics of the top 10 CAEs in 89 AcMADS-box gene promoters. Detailed information for each CAE is listed in Supplementary Table S5.

The 2 kb promoter sequences of 89 AcMADS-box genes were isolated from the genome of kiwifruit, and the cis-acting elements (CAEs) were predicted by PlantCARE. A total of 2204 CAEs are divided into 52 types and 19 functional modules. (Figures 5A–C and Supplementary Table S5). The AcMADS-box genes contain 2 (*AcMADS24*) to 53 (*AcMADS47*) CAEs, which were mainly associated with light (49.73%), phytohormone responsiveness (ABA 9.17%, MeJA 8.98%, GA 5.72%, SA 2.68%, and ZM 1.36%), anaerobic induction (8.85%) and abiotic stress (drought 2.45%, defense and stress 1.97%, and low-temperature 1.86%) (Figure 5D). Light responsiveness-related CAEs distributed on each gene promoter of 89 AcMADS-box genes, while flavonoid biosynthetic genes regulation, palisade mesophyll cells, and cell cycle regulation-related CAEs only distributed on 7 to 8 AcMADS-box gene promoters (Supplementary Table S5). Among the 52 types of CAEs, Box 4 had the largest number of 251, and GATT-motif, chs-CMA2b, and CAG-motif had the least number of 1, respectively (Figure 5E and Supplementary Table S5). In addition, the CAEs related to light response are the most, such as Box 4, G-box, GT1-motif, TCT-motif, GATA-motif, I-box, AE-box, MRE, and TCCC-motif. The P-box, TATC-box, TGA-box, TGA-element, and GARE-motif were related to GA-responsiveness. The CGTCA-motif and TGACG-motif were associated with MeJA-responsiveness. The ABRE and ARE were involved in ABA-responsiveness and anaerobic induction, respectively (Supplementary Table S5). Those results showed that AcMADS-box genes mainly respond to environmental changes, hormone response and organ development in kiwifruit.

### 3.6 Gene dupulacation analysis of kiwifruit MADS-box genes

**FIGURE 6:**
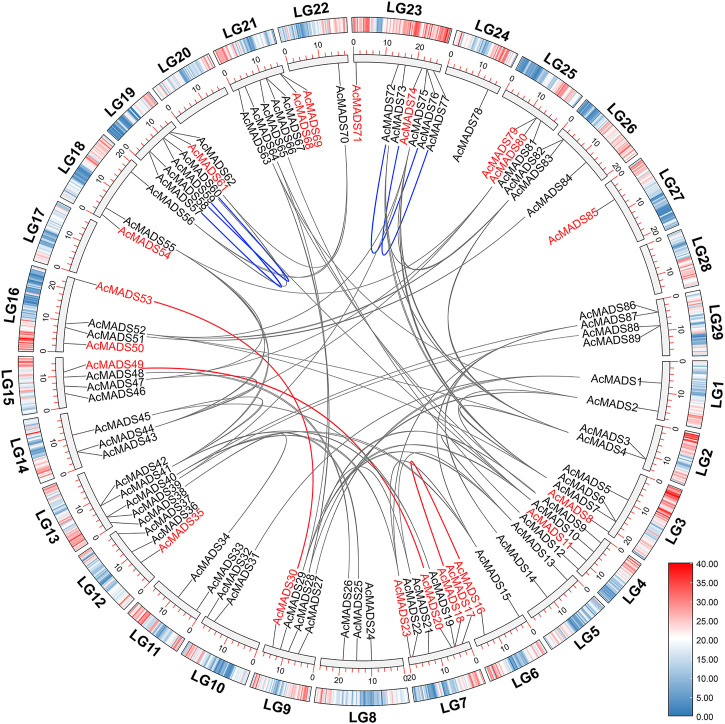
Synteny analysis of AcMADS-box genes in A. chinensis. The genes name in red and black represent type I and type II AcMADS-box genes, respectively. Gray and red lines represent inter-chromosomal synteny blocks in type I and type II AcMADS-box genes, respectively. The blue lines indicate the collinearity inside the same chromosome. The colors from blue to red on every chromosome of the outside boxes indicate gene density.

Collinearity analysis of AcMADS-box genes was performed using MCScanX. The results showed that 62 out of 89 AcMADS-box genes had collinearity between and within chromosome in kiwifruit. Among the 62 AcMADS-box genes, most of them have 1 to 4 pairs of duplicated segments, while *AcMADS6* has the largest number of pairs, with 6 (Supplementary Table S6). A total of 68 pairs of collinearity were distributed on 24 chromosomes, of which LG23 had the most collinearity (13 pairs), whereas no AcMADS-box gene collinearity was found in LG17, LG20, LG24, LG27, and LG28 (Figure 6). Most AcMADS-box genes collinearity are inter-chromosomal duplications, and only four pairs of collinearity of type II AcMADS-box genes (*AcMADS57/AcMADS59*, *AcMADS58/AcMADS60*, *AcMADS72/AcMADS76*, and *AcMADS73/AcMADS77*, the blue lines) are intra-chromosomal duplications (Figure 6). Meanwhile, only 3 pairs of collinearity (*AcMADS16/AcMADS17*, *AcMADS20/AcMADS49*, and *AcMADS30/AcMADS53*, the red lines) belong to type I AcMADS-box genes, while the remaining 65 pairs belong to type II AcMADS-box gene (Figure 6). This may further explain why the number of type II AcMADS-box (68 genes) in kiwifruit is significantly higher than that in type I (21 genes).

### 3.7 Expression profiles of kiwifruit MADS-box genes in different tissues

**FIGURE 7:**
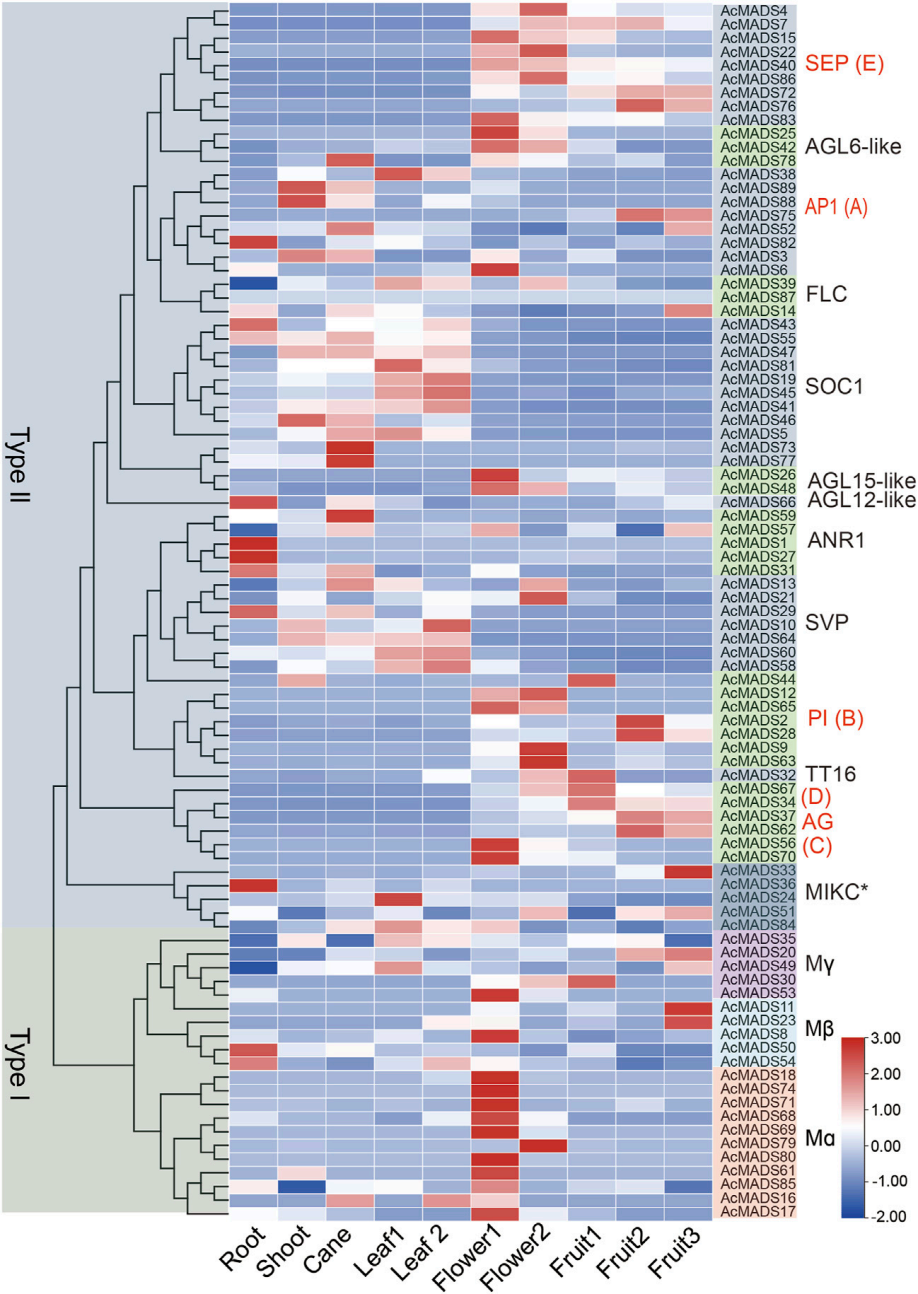
Expression profiles of AcMADS-box genes in ten different tissues of kiwifruit. Leaf 1, source leaf; Leaf 2, sink leaf; Flower 1, flower bud; Flower 2, opening flower; Fruit 1/2/3, fruit of 20, 147, and 224 days after anthesis. The phylogenetic tree and the subgroup of AcMADS-box genes were on the left and right sides, respectively. The expression levels of AcMADS-box genes from high to low are represented by a heat-map from red to green. Details on expression levels are presented in Supplementary Table S7.

To explore the function of AcMADS-box genes, the expression profiling of 89 AcMADS-box genes were determined by reanalyzing the publicly published RNA-Seq data in 10 different tissues, including root, shoot, cane, source leaf (leaf 1), sink leaf (leaf 2), flower bud (flower 1), opening flower (flower 2), the fruit of 20 days after anthesis (DAA) (fruit 1), 147 DAA (fruit 2) and 224 DAA (fruit 3) (Supplementary Table S7). We found that 88 AcMADS-box genes have diverse expression patterns in different tissues, whereas no expression of the *AcMADS87* gene was detected (Figure 7). The heat-map showed that the same subgroup in type II have similar expression patterns. For example, the *SEP, PI, TT16, AG*, and *AGL15-like* subgroups were expressed significantly in flowers and fruits, while the *SOC1* and *AGL12-like* subgroups were expressed significantly in vegetative organs (Figure 7). Notably, most genes had higher expression levels in flower, and some genes are expressed in specific tissues. For example, *AcMADS1, AcMADS27, AcMADS36*, and *AcMADS82* were highly expressed in the root; *AcMADS73, AcMADS77*, and *AcMADS59* were highly expressed in the cane; *AcMADS2, AcMADS28, AcMADS33, AcMADS11*, and *AcMADS23* were highly expressed in the fruit (Figure 7). Those results indicated that different subgroups of AcMADS-box genes may regulate different organ development in kiwifruit.

Moreover, many ABCDE model homologous genes of Kiwifruit were highly expressed in flowers. For example, the AP1 (A) type member *AcMADS6*, the *PI* (B) type members *AcMADS9/12/63/65*, the *AG* (C/D) type members *AcMADS56/70*, and the *SEP* (E) type members *AcMADS4/15/22/40/83/86* have high expression levels in flower tissues as expected (Figure 7). Those genes may function as the ABCDE model-related genes in kiwifruit that regulate floral organ differentiation. Interestingly, 10 out of the 11 Mα subgroup AcMADS-box genes were also expressed significantly higher in flowers (Figure 7). It can be speculated that the Mα subgroup AcMADS-box genes may also be involved in the regulation of kiwifruit flower development.

### 3.8 Expression patterns of ABCDE model-related AcMADS-box genes in male and female flowers of kiwifruit

**FIGURE 8:**
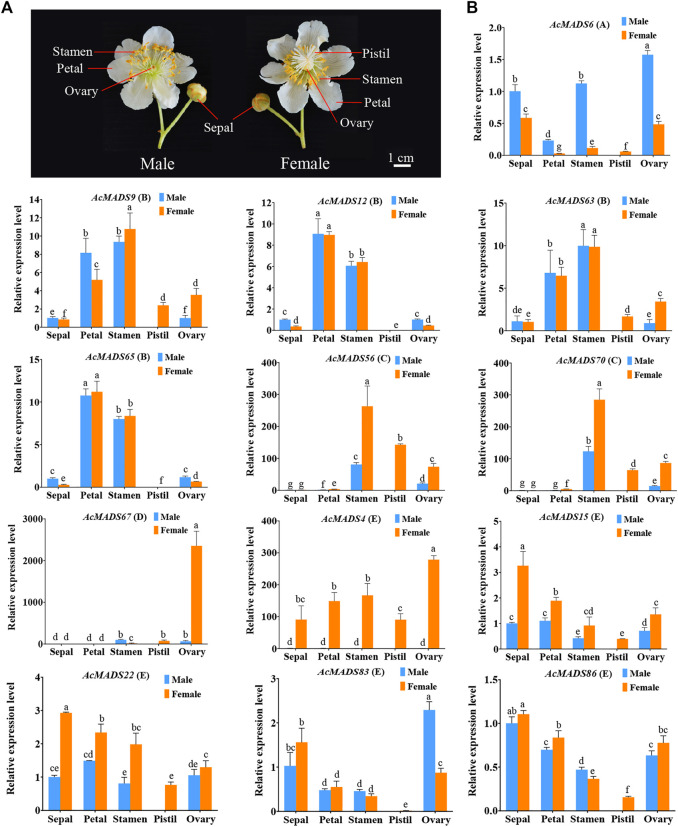
Expression patterns of floral organs development ABCDE model-related AcMADS-box genes in male and female flowers of kiwifruit. **(A)** Structure of male and female flowers of kiwifruit. The sepal, petal, stamen, and degenerated ovary of male flowers, and sepal, petal, aborted stamen, pistil and ovary of female flowers. **(B)**. The qRT-PCR analysis of floral organ ABCDE model-related AcMADS-box genes in kiwifruit male and female flowers. *AcMADS6* belongs to type A; *AcMADS9, AcMADS12, AcMADS63*, and *AcMADS65* belong to type B; *AcMADS56 and AcMADS70* belong to type C; *AcMADS67* belong to type D; *AcMADS4, AcMADS15, AcMADS22, AcMADS83*, and *AcMADS86* belong to type E. The data were analyzed for significance of differences by one-way ANOVA test (*p* < 0.05). Bars with the same small letters are not significantly different, and bars that do not share the same small letters are significantly different from each other.

According to the expression profiles of AcMADS-box genes, 13 ABCDE model-related genes that highly expressed in flower were selected for qRT-PCR analysis. The expression patterns of most genes fit the ABCDE model classification. For example, the expression patterns of four B-type genes (*AcMADS9, AcMADS12, AcMADS63*, and *AcMADS65*) were consistently, and mainly expressed in petals and stamens; the D-type gene *AcMADS67* was significantly expressed in the female ovary (Figure 8). However, some genes are also expressed in other tissues. *AcMADS6*, an A-type AcMADS-box gene, was highly expressed in the stamen and ovary besides sepals; *AcMADS56 and AcMADS70*, two C-type genes, were expressed not only in stamens and carpels but also in the ovary (Figure 8). These results suggest that some A-type and C-type genes may also be involved in regulating ovary development in kiwifruit. In addition, E-type AcMADS-box genes were expressed in all tissues, and the expression level in the pistil was the lowest (Figure 8). In male and female flowers of kiwifruit, *AcMADS6* was mainly expressed in male flowers, while C-type, D-type and E-type genes were mainly expressed in female flowers. (Figure 8). In addition, the expression level of *AcMADS67* in the female ovary is significantly higher than that in other tissues, including the male ovary (Figure 8), which may be related to the degradation of male embryos. It is noteworthy that the expression level of *AcMADS4, AcMADS56*, and *AcMADS70* in female flowers were significantly higher than those in male flowers (Figure 8). Moreover, the promoter cis-element analysis of two key sex differentiation regulators of kiwifruit, *SyGl* and *FrBy*, showed that the promoter of *SyGl* contained 28 MADS-box binding sites, while the promoter of *FrBy* did not (Supplementary Table S8). These results indicated that *AcMADS4, AcMADS56*, and *AcMADS70* may be involved in flower sex differentiation of kiwifruit by binding to the promoter of *SyGl* gene.

## 4 Discussion

MADS-box gene family are ubiquitous in plants and have been identified in more than 40 species, such as Arabidopsis (109 genes) (Kofuji et al., 2003), rice (75 genes) (Arora et al., 2007), cucumber (43 genes) (Hu and Liu, 2012), soybean (163 genes) (Fan et al., 2013), peach (79 genes) (Wells et al., 2015), chinese cabbage (167 genes) (Saha et al., 2015), banana (96 genes) (Liu et al., 2017), pineapple (48 genes) (Zhang et al., 2020), maize (75 genes) (Zhao D. et al., 2021), musa balbisiana (97 genes) (Lakhwani et al., 2022). In this study, 89 MADS-box genes were identified in kiwifruit, which is the same as foxtail millet (89 genes) (Lai et al., 2022). The variation in the number of MADS-box genes among species may be due to whole genome duplication (WGD) events during evolution. For example, rice has experienced a recent WGD, whereas cucumber and pineapple have only experienced ancient WGDs, resulting in less MADS-box genes than rice (Arora et al., 2007; Huang et al., 2009; Zhang et al., 2020; Guan et al., 2021).

In most species, the number of type II MADS-box genes is greater than that of type I, such as rice (32 of type I, 43 of types II) (Arora et al., 2007), chinese cabbage (62 of type I, 105 of types II) (Saha et al., 2015) and populus (41 of type I, 60 of types II) (Leseberg et al., 2006). In kiwifruit, the number of type II (68 genes) AcMADS-box is more than three times that of type I (21 genes), which may be attributed to the more duplication events of type II genes in kiwifruit. Gene collinearity analysis of AcMADS-box genes showed that only 3 pairs of collinearity belong to type I AcMADS-box genes, the remaining 65 pairs all belong to type II. On the whole, type II AcMADS-box genes have longer length of the gDNA, more complex gene structures, and more number of protein domains in kiwifruit. It is indicated that type II AcMADS-box genes may have more complex gene functions. It is noteworthy that the Mα subgroup AcMADS-box genes of type I has a maximum gene number of 11, which is consistent with the maximum gene members of 24 in Arabidopsis (Abdullah-Zawawi et al., 2021). In addition, although Mα subgroup AcMADS-box genes do not belong to the ABCDE model, 10 out of the 11 Mα subgroup genes were significantly higher in kiwifruit flowers. It can be speculated that the Mα subgroup AcMADS-box genes may also be involved in the regulation of kiwifruit flower development, which is different from other species and deserves further study.

The gene functions of the MADS-box family are diverse, and some AcMADS-box gene functions have been identified in kiwifruit. For example, *AcFLCL* (*AcMADS14*), an homologous to Arabidopsis *FLOWERING LOCUS C* (*FLC*) is introduced by low-temperature and plays an important role in activating growth (Mitalo et al., 2019; Voogd et al., 2022). Overexpression of *AcFLCL* in kiwifruit caused early bud breaking, whereas CRISPR-Cas9 editing of the *AcFLCL* gene caused delayed bud breaking (Voogd et al., 2022). In this study, the expression profile analysis showed that *AcMADS14* was highly expressed in fruit, and it may also be involved in fruit development in kiwifruit. In general, most proteins in the same subgroup of MADS-box family have similar functions due to containing the same motifs, such as *FLC*, *AGL15-like*, *AG*, and *SVP* subgroups. *SHORT VEGETATIVE PHASE* (*SVP*) is involved in the regulation of flowering and bud dormancy in Arabidopsis (Lee et al., 2007; Tao et al., 2012). In kiwifruit, *SVP1* (*AcMADS29*), *SVP2* (*AcMADS10*), *SVP3* (*AcMADS64*), and *SVP4* (*AcMADS60*) accumulated significantly in buds in winter, and were down-regulated before floral differentiation, suggesting that these genes may promote bud dormancy and inhibit flower bud differentiation (Wu et al., 2014). Further studies showed that *AcSVP2* inhibited bud growth through the ABA pathway (Wu et al., 2017; Wu et al., 2019) and *AcSVP3* inhibits flowering in kiwifruit (Wu et al., 2014). Here, we have identified three new *SVP* genes, *AcMADS13*, *AcMADS21*, and *AcMADS58*. The gene expression pattern of *AcMADS58* is consistent with that of *SVP3* and *SVP4*, both of them are mainly expressed in vegetative organs, and they may have functional redundancy. However, *AcMADS13* and *AcMADS21* are mainly expressed in flowers, and they may be involved in the regulation of flowering. Among the type II AcMADS-box genes, the *SOC1* subgroup contains the largest number of genes. Nine *SUPPRESSOR OF OVEREXPRESSION OF CONSTANS1* (*SOC1*) genes, named *AcSOC1a* to *AcSOC1i* (*AcMADS47*, *81*, *55*, *43*, *41*, *19*, *45*, *5*, and *46* of this study, respectively), have been reported to promote flowering (Voogd et al., 2015). Here, two new *SOC1-like* genes *AcMADS73* and *AcMADS77* were identified. They were highly expressed in cane and belonged to a new branch of the *SOC1* subgroup in the evolutionary tree, which may imply their potential function in the development of cane in vine plants.

In some dioecious plants, MADS-box family genes are closely related to the differentiation of male and female flowers. For example, *LcMADS51* may be involved in the formation of litchi carpel, and six MADS-box genes including *LcMADS42*/*46*/*47*/*75*/*93*/*100* may play a role in stamen development (Guan et al., 2021). In *Momordica dioica* Roxb, *MdMADS12*/*13*/*14*/*15* regulate male gametophyte development, while *MdMADS16* and *MdMADS17* play important roles in female gametophyte formation and seed development (Mohanty and Joshi, 2018). In *Cunninghamia lanceolata*, MADS-box genes including *GGM7*, *SVP,* and *AGL15* are candidate genes for sex-specific development of cones due to their specific expression in female/male cones (Wang et al., 2020). In kiwifruit, a total of 9 ABCDE model-related AcMADS-box genes (2 of A-type, 3 of B-type, 1 of C-type, and 3 of E-type) were identified and ectopic overexpressed in Arabidopsis suggesting that they are required for floral organ specification (Varkonyi-Gasic et al., 2011). In this study, 21 new ABCDE model-related genes were isolated, including 3 type D genes (*AcMADS34*, *AcMADS37*, and *AcMADS67*). Interestingly, these three genes were mainly expressed in fruits rather than flowers, suggesting functional differentiation of class D genes in kiwifruit. Furthermore, 13 genes of the ABCDE model were selected for qRT-PCR analysis. As expected, four B-type genes (*AcMADS9*, *AcMADS12*, *AcMADS63*, and *AcMADS65*) were mainly expressed in petals and stamens, and one D-type gene *AcMADS67* was significantly expressed in the female ovary. However, A-type genes *AcMADS6* and C-type genes *AcMADS56*/*70* also had high expression levels in the ovary, indicating that A-type and C-type genes may also be involved in ovary development in kiwifruit. Notably, the expression levels of *AcMADS4*, *AcMADS56*, and *AcMADS70* in female flowers were significantly higher than those of male flowers, especially *AcMADS4*. Moreover, the promoter of *SyGl* gene contained 28 MADS-box binding sites and some AcMADS-box genes, such as *AcMADS4*, *AcMADS56*, and *AcMADS70*, may regulate the development of pistils by binding to the promoter of *SyGl* gene, thereby regulating the sex differentiation of kiwifruit, which is need to be further studied.

## 5 Conclusion

In this study, 89 AcMADS-box genes were isolated from *A. chinensis* Red 5 genome, and their chromosome position, gene structure, protein conserved domain, motif, and expression profile were analyzed. Then, the expression levels of floral organ ABCDE model-related AcMADS-box genes in different parts of male and female flowers were measured. The expression levels of *AcMADS4*, *AcMADS56*, and *AcMADS70* in female flowers were significantly higher than those in male flowers. The promoter of *SyGl* contains several MADS-box binding sites. *AcMADS4*, *AcMADS56*, and *AcMADS70* may be involved in floral sex differentiation by binding to the promoter of *SyGl*. This study will help us to comprehensively understand the characteristics of the kiwifruit MADS-box gene family and screen for AcMADS-box genes involved in kiwifruit sex differentiation.

## Data Availability

The original contributions presented in the study are included in the article/Supplementary Material, further inquiries can be directed to the corresponding authors.

## Supplementary material

Supplementary Figure S1 Number of *A. chinensis* MADS-box genes on each chromosome.

Supplementary Figure S2 Number of *A. chinensis* MADS-box genes on each subgroup.
