# Supplementary figures and images for: Genome-wide analysis of MADS-box gene family in kiwifruit (Actinidia chinensis var. chinensis) and their potential role in floral sex differentiation
Source: Front Genet. 2022 Nov 17;13:1043178. doi: 10.3389/fgene.2022.1043178 (PMC9714460; doi:10.3389/fgene.2022.1043178)

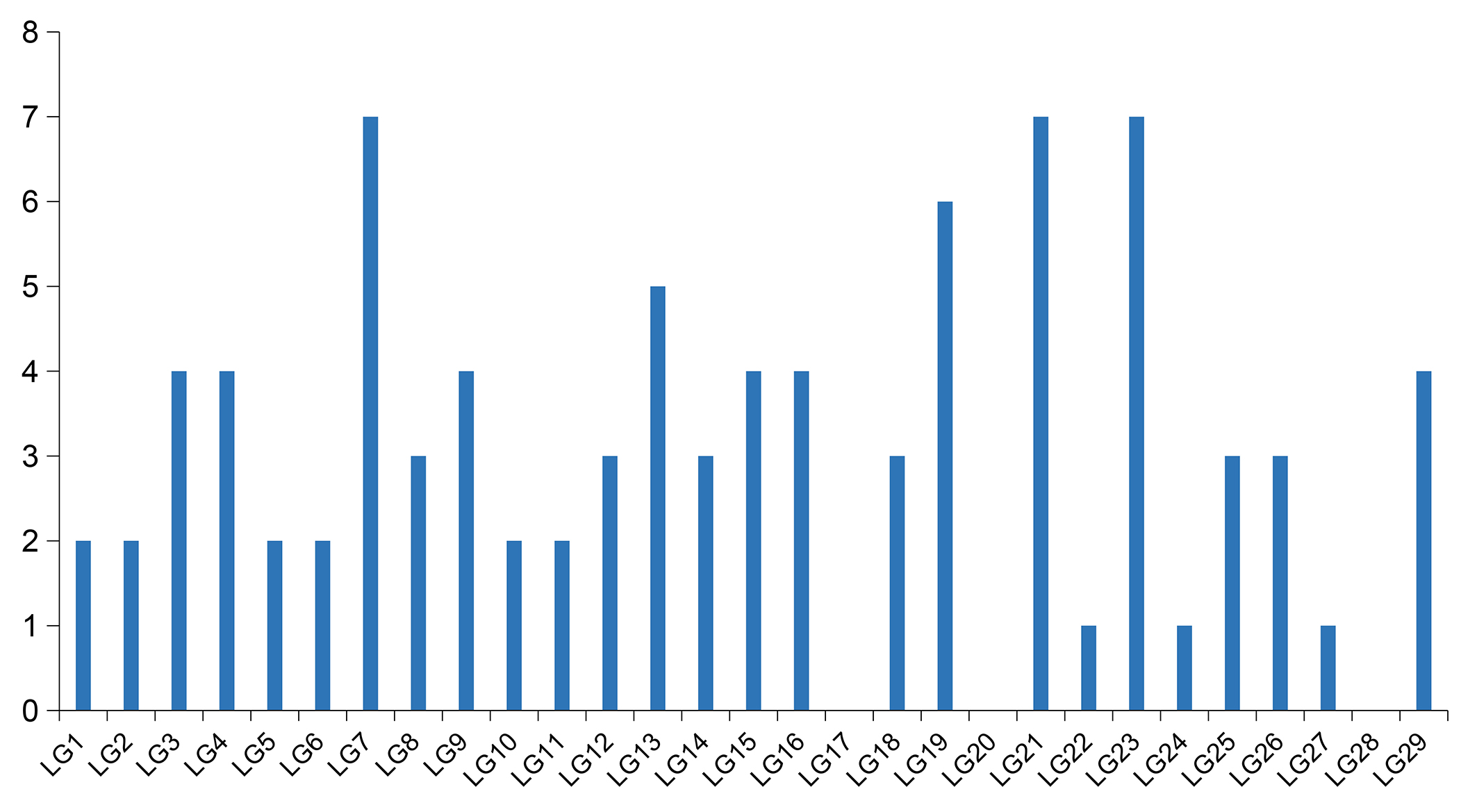

Supplement: Supplementary file 1 [file Image1.JPEG]

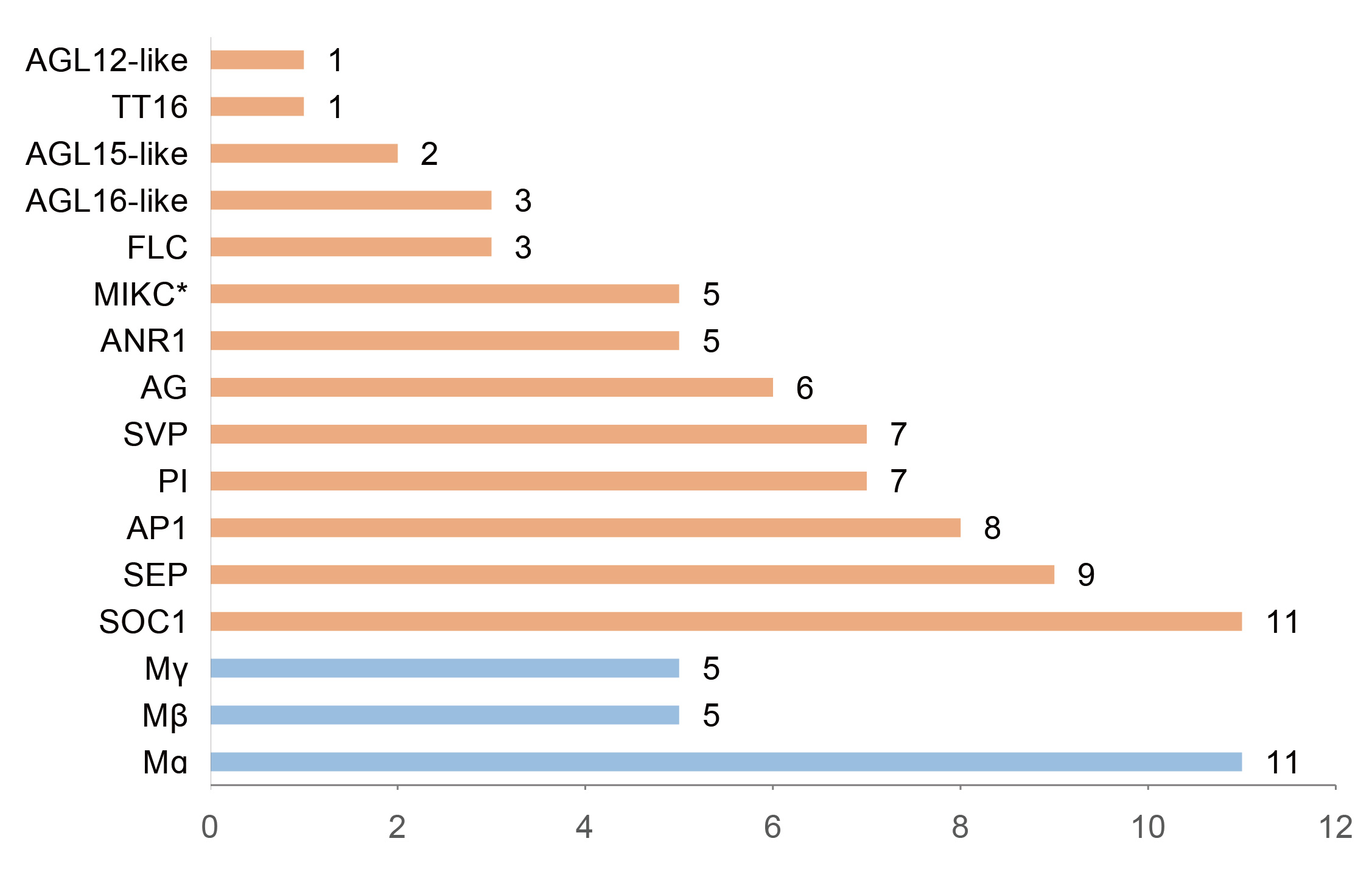

Supplement: Supplementary file 2 [file Image2.JPEG]
